# Case Report: Myoclonic and tremulous movements associated with COQ8A-related coenzyme Q10 deficiency type 4

**DOI:** 10.3389/fgene.2025.1682085

**Published:** 2026-01-07

**Authors:** Di Wang, Guojian Zhang, Xiaojing Fang, Fang Liu, Li Wang

**Affiliations:** Department of Neurology, The First Hospital of Tsinghua University, Beijing, China

**Keywords:** *COQ8A* gene, primary coenzyme Q10 deficiency, myoclonic tremor, mitochondrial disorders (MIDs), movement disorder

## Abstract

**Background:**

Primary coenzyme Q10 (CoQ10) deficiency is a rare, treatable mitochondrial disorder often caused by biallelic pathogenic variants in *COQ8A* gene (also known as *ADCK3*). It typically manifests as childhood-onset cerebellar ataxia with variable multisystem involvement. Early recognition is critical, as CoQ10 supplementation has potential to significantly alleviate clinical manifestations and modify natural progression of the disease. Here, we provide a rare phenotype of paroxysmal dyskinesias caused by compound heterozygous variants of *COQ8A* gene.

**Case:**

A 21-year-old man presented with myoclonic tremor, mild dysarthria, ataxia and emotional instability. The brain MRI showed cerebellar atrophy. Biochemical workup revealed low plasma CoQ10 levels. Whole-exome sequencing identified compound heterozygous *COQ8A* variants: two novel missense substitutions [NM_020247.5:c.641T>A (p.Leu214Gln), NM_020247.5:c.1621T>C (p.Ser541Pro)], each inherited from an asymptomatic parent. The patient was initiated on oral CoQ10 at a dose of 200 mg twice daily, accompanied by supportive interventions targeting emotional regulation. A marked improvement in tremor symptoms was observed shortly after treatment initiation; however, intermittent muscle tremors persisted during periods of emotional agitation. At 1-year follow-up, the patient remained on CoQ10 at 300 mg twice daily and levetiracetam at 500 mg twice daily, with sustained symptom control.

**Conclusion:**

This case highlights that *COQ8A*-related CoQ10 deficiency can present with serious neurological crises among young people and underscores the importance of rapid genetic diagnosis in such scenarios. Early and aggressive CoQ10 supplementation led to clinical stabilization in our patient, reinforcing that primary CoQ10 deficiency is a reversible cause of neurodegenerative disease. We emphasize genotype-phenotype diversity in *COQ8A* disease and the crucial need for early detection and treatment to improve prognosis. We propose that clinicians maintain a high index of suspicion for primary CoQ10 deficiency in patients presenting with unexplained dystonia or ataxia, as timely intervention may significantly improve clinical outcomes.

## Introduction

Coenzyme Q10 (CoQ10), or ubiquinone, is a lipid-soluble cofactor essential for mitochondrial energy production and cellular redox homeostasis. It shuttles electrons from complexes I and II to complex III in the respiratory chain and also serves as a potent antioxidant and a cofactor in pyrimidine biosynthesis. Primary CoQ10 deficiency is an autosomal recessive mitochondrial disorder caused by pathogenic variants in any of the genes encoding enzymes of CoQ10 biosynthetic pathway (Cotta et al., 2020). *COQ8A* (archaically known as *ADCK3* or *CABC1*) is one such gene; it encodes an atypical protein kinase required for effective CoQ10 biosynthesis in mitochondria. Biallelic *COQ8A* mutations cause primary CoQ10 deficiency type 4 (COQ10D4), also termed *COQ8A*-ataxia (Traschütz et al., 2020). Since the first description in 2007, over 100 cases of *COQ8A* deficiency have been reported worldwide. This condition most frequently presents as a childhood-onset cerebellar ataxia (often an autosomal recessive cerebellar ataxia, ARCA) (Paprocka et al., 2022). Cerebellar atrophy on neuroimaging is a common hallmark. However, the clinical phenotype is remarkably heterogeneous. In addition to progressive ataxia, patients can exhibit developmental delay, intellectual disability, hyperkinetic movement disorders (dystonia, tremor), epilepsy, psychiatric symptoms, and migraines (Paprocka et al., 2022). Multisystem involvement such as ophthalmoplegia or ptosis, sensorineural hearing loss, myopathy, cardiomyopathy, and nephrotic syndrome has also been described (Cotta et al., 2020). The disease severity and organ involvement can vary even among patients with the same genotype, indicating that other genetic or environmental factors modulate the phenotype.

A striking feature of primary CoQ10 deficiencies is their potential treatability. Unlike many mitochondrial diseases, CoQ10 deficiency can often be substantially improved or even reversed with high-dose CoQ10 supplementation, especially if therapy is initiated early (Lopriore et al., 2024). Case reports and series have documented improvements in ataxia, exercise tolerance, and seizures after months of CoQ10 therapy (Zhang et al., 2020). However, treatment responses are variable: while some patients experience marked neurological improvement, others exhibit only disease stabilization or progressive deterioration despite CoQ10 supplementation (Ramesh et al., 2023). The efficacy of treatment appears to depend on multiple factors including the patient’s age at treatment initiation, disease duration, the nature of the *COQ8A* mutations, and possibly the bioavailability of the CoQ10 formulation used. Genotype may also play a role: recent cohort analyses suggest that patients with biallelic loss-of-function variants of *COQ8A* tend to have a pure ataxia phenotype that may be more responsive to CoQ10, whereas those with certain missense mutations can have more complex multisystem disease (Chavira-Hernández et al., 2023).

Here we present a new case of primary CoQ10 deficiency due to compound heterozygous *COQ8A* mutations, in which the patient suffered truncal myoclonic movements, mild dysarthria and ataxia. This case underscores the importance of considering a treatable mitochondrial disorder in dystonia and ataxia syndromes, and of deploying rapid genetic testing in critical illness. We provide a detailed case report of the clinical presentation, diagnostic workup including whole-exome sequencing (WES) and biochemical studies, and the patient’s response to CoQ10 therapy. By integrating our case findings with published data, we highlight current challenges and recommendations in the diagnosis and management of *COQ8A*-related CoQ10 deficiency.

## Case presentation

### Patient history

A 21-year-old Asian male presented with myoclonic tremor, mild dysarthria and ataxia. The symptom consisted of semi-continuous myoclonic and tremulous movements, mainly affecting the upper limbs and trunk, with occasional propagation to the left abdominal wall. These movements fluctuated in amplitude and were exacerbated by emotional stress, fatigue, or vigorous physical activity, but were not triggered by sudden voluntary movement or external stimuli, distinguishing them from classical paroxysmal kinesigenic dyskinesia. Each exacerbation lasted several minutes to hours and was followed by partial recovery without loss of consciousness. He was born to non-consanguineous parents with no significant family history of neurological disorders. During childhood, the patient exhibited mildly delayed language development. He had achieved independent walking but was unstable by 3 years of age. There was no history of seizures, visual changes, hearing loss, or muscle weakness during childhood.

### Physical and laboratory findings

**FIGURE 1:**
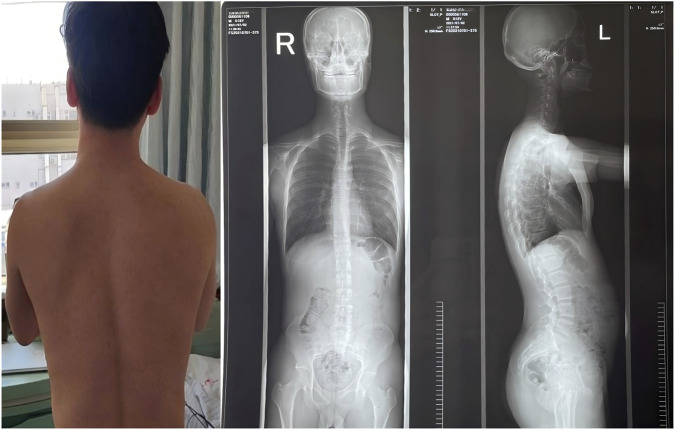
Full-length standing anteroposterior and lateral X-ray images of the spine. The thoracic spine exhibits a mild rightward curvature centered at T7, while the lumbar spine demonstrates a mild leftward curvature centered at L3. No obvious abnormalities were observed in the cervical and sacrococcygeal spine.

**FIGURE 2:**
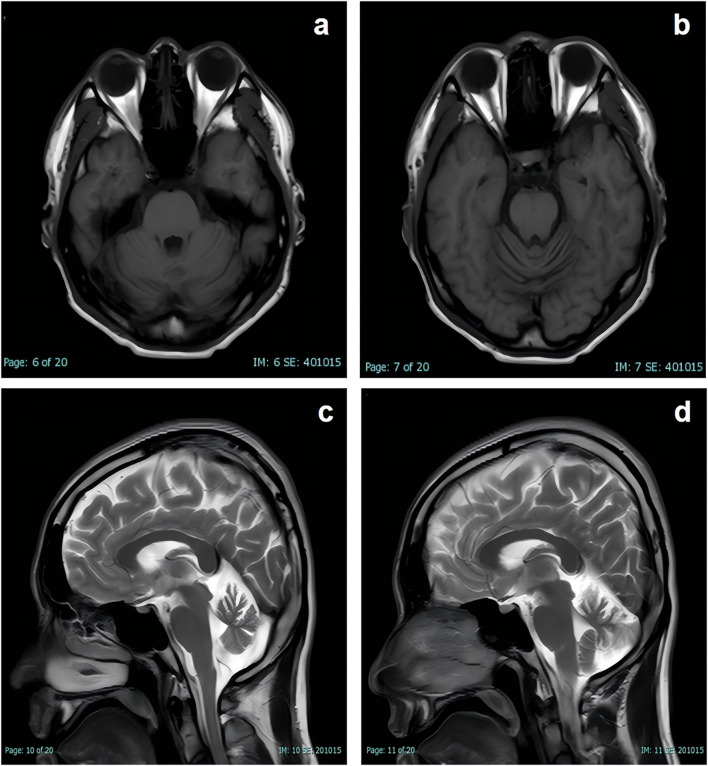
Brain MRI shows marked cerebellar atrophy. **(a,b)** Axial T1-weighted images demonstrate reduced cerebellar volume; **(c,d)** sagittal T2-weighted images further delineate cerebellar folia thinning.

Neurological examination revealed fine myoclonic tremor in both upper limbs and intermittent truncal jerks, more prominent on the left side. No sustained dystonia or chorea was observed. The patient was alert and conscious, with mild dysarthria. Higher cortical functions were intact. Cranial nerve examination revealed no abnormalities. Limb muscle strength was grade V, with normal muscle tone. Scoliosis deformity was observed (Figure 1). Muscle twitching in the left mid-to-lower abdomen was noted, accompanied by torsional movements of the trunk (video). Deep tendon reflexes were symmetrically elicited in all limbs. Bilateral finger-to-nose tests were steady and accurate. Heel-to-shin testing of both lower extremities was mildly impaired in stability and accuracy. The Romberg sign was negative, though the patient demonstrated difficulty with tandem gait. Pathological reflexes were absent, and no further neurological abnormalities were identified. Cardiovascular and respiratory exams were unremarkable. Cranial MRI demonstrated mild cerebellar atrophy, with marked atrophic changes evident in the sagittal view (Figure 2). MRI-Brain DTI (non-contrast) showed that the bilateral corticospinal tracts were normally aligned, without obvious interruption or displacement. Cerebellar fiber tracts were slightly reduced. Fractional anisotropy (FA) values indicated that the FA of the right cerebellar hemisphere was slightly lower than normal (normal range: ≥0.18).

**FIGURE 3:**
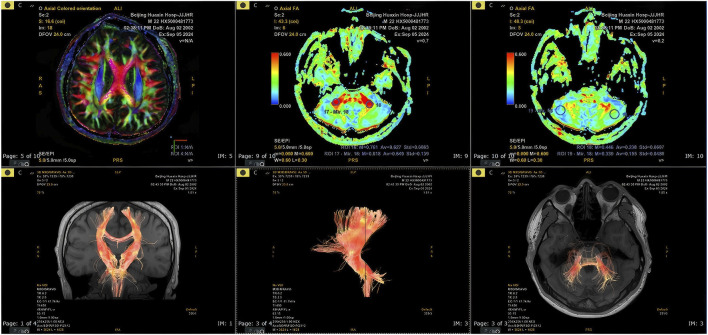
MRI-Brain DTI showed that the bilateral corticospinal tracts were normally aligned, without obvious interruption or displacement. Cerebellar fiber tracts were slightly reduced. Fractional anisotropy (FA) values indicated that the FA of the right cerebellar hemisphere was slightly lower than normal (normal range: ≥0.18). SPECT cerebral perfusion imaging revealed mildly heterogeneous blood flow in the frontal and parietal lobes.

**FIGURE 4:**
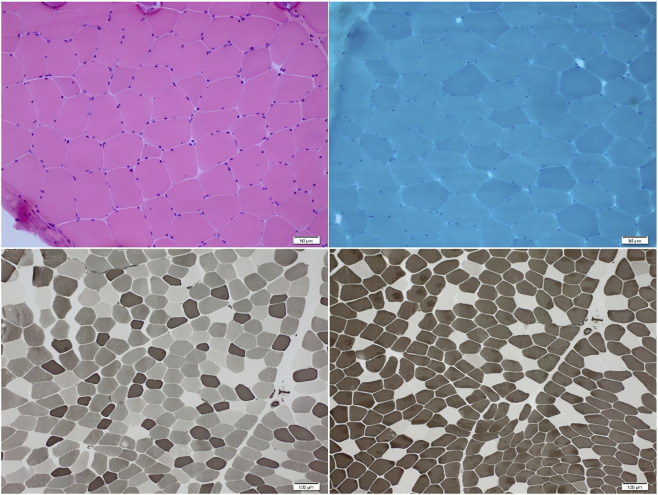
Skeletal muscle biopsy revealed no significant abnormalities.

SPECT cerebral perfusion imaging revealed mildly heterogeneous blood flow in the frontal and parietal lobes (Figure 3). Muscle biopsy of the left biceps brachii revealed that the primary pathological finding was the presence of occasional atrophic muscle fibers. The changes were mild and lacked disease-specific features. There were no pathological findings characteristic of inflammatory myopathies, muscular dystrophies, or neurogenic skeletal muscle damage (Figure 4). Needle electromyography (EMG) revealed no evidence of neurogenic or myogenic damage. No fibrillation potentials or positive sharp waves were observed in the paraspinal (L4–5 level), sternocleidomastoid, deltoid, abductor pollicis brevis, tibialis anterior, or vastus medialis muscles. Motor unit potentials were of normal duration with mixed interference pattern during maximal contraction. These findings excluded peripheral neuromuscular pathology and supported a central origin of the movement disorder. There were no signs of cardiomyopathy on echocardiogram and no organomegaly. Laboratory studies showed serum creatine kinase, alpha-fetoprotein, vitamin E level, and very long chain fatty acids were normal. Notably, the plasma CoQ10 level was low at 0.31 μmol/L (reference range ∼0.46–1.85), suggesting CoQ10 deficiency. A lumbar puncture found normal cerebrospinal fluid (CSF) cell counts and protein.

## Genetic analysis

**FIGURE 5:**
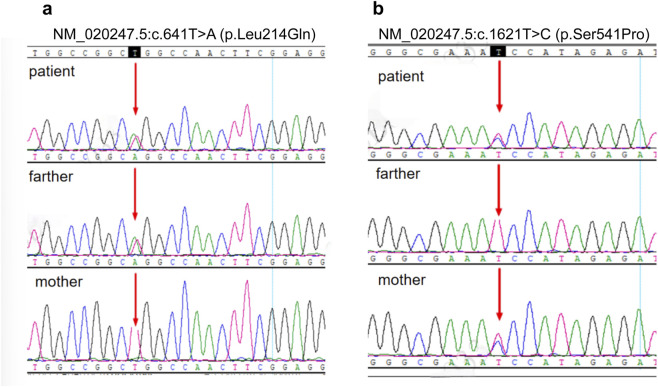
Sanger sequencing validation of compound heterozygous mutations in the *COQ8A* gene. **(a)** The NM_020247.5:c.641T>A (p.Leu214Gln) variant demonstrates a thymine-to-adenine substitution at nucleotide position 641 (indicated by red arrow). The patient and the father are heterozygous for this variant, while the mother does not carry it. **(b)** The NM_020247.5:c.1621T>C (p.Ser541Pro) variant shows a thymine-to-cytosine substitution at position 1,621 (indicated by red arrow). The patient and the mother are heterozygous for this variant, whereas the father does not carry it.

We performed rapid whole-exome sequencing (WES) on patient’s blood DNA, with targeted analysis of genes associated with mitochondrial disease and dystonia disease. This revealed compound heterozygous missense variants in the *COQ8A* gene (NM_020247.5):c.641T>A (p.Leu214Gln) and c.1621T>C (p.Ser541Pro), each inherited from an asymptomatic parent. Both variants are absent from population databases (gnomAD frequency = 0) and have not been previously reported in the literature. Each substitution affects a highly conserved residue within the kinase-like domain of the *COQ8A* protein and is predicted to be deleterious by multiple *in silico* algorithms. According to ACMG criteria, both variants were initially classified as variants of uncertain significance (VUS), but the combined evidence-segregation in trans, phenotype concordance, and biochemical response supports classification as likely pathogenic. These *COQ8A* missense change is extremely rare (absent from gnomAD) and affects a highly conserved amino acid in the kinase-like domain of *COQ8A*. Our patient’s variants are parallel missense affecting the identical residue (Leu214 and Ser541), thus considered likely pathogenic by homology. Segregation analysis by Sanger sequencing showed that the mutation (p.Leu214Gln) was inherited from his father and the p.Ser541Pro from his mother (Figure 5), confirming these variants were in trans (compound heterozygous). Both parents are healthy and neurologically normal, consistent with autosomal recessive inheritance.

**FIGURE 6:**
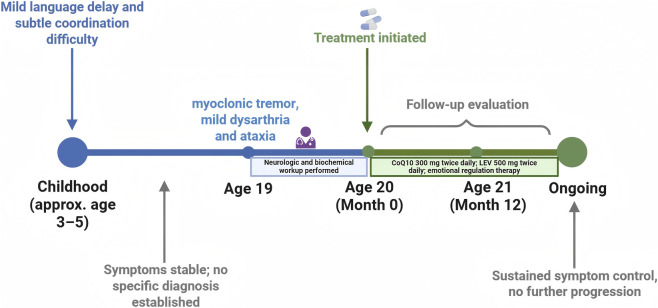
Timeline of clinical course.

According to the American College of Medical Genetics and Genomics (ACMG) criteria, both COQ8A variants were initially classified as variants of uncertain significance (VUS) due to the absence of prior reports. However, integrated evidence from segregation analysis, clinical phenotype correlation, biochemical findings, and therapeutic response strongly supports their reclassification as likely pathogenic variants contributing to the patient’s primary CoQ10 deficiency. First, *COQ8A* is the only gene in which the patient harbored two rare protein-altering variants, matching the expected recessive cause. Second, the patient’s phenotype aligns with *COQ8A*-related disease: Adolescence-onset cerebellar ataxia progressing to paroxysmal dyskinesias with low CoQ10 levels (Lopriore et al., 2022). Third, the affected Leu214 and Ser541 is highly conserved across species and lies within a functionally important region, and significant clinical improvement was observed after initiation of COQ10 supplementation. *COQ8A*-related primary CoQ10 deficiency is autosomal-recessive, with disease caused by biallelic pathogenic variants; both homozygous and compound-heterozygous configurations have been described in cohort studies and case series (Traschütz et al., 2020). In our patient, segregation analysis, phenotype–genotype correlation, markedly reduced plasma CoQ10, and favorable treatment response collectively support the variants’ likely pathogenicity. All these prior findings suggest that the compound *COQ8A* variants are highly suggestive of primary CoQ10 deficiency type 4 (COQ10D4), supported by the patient’s clinical phenotype, biochemical findings, and treatment response. No other pathogenic variants were identified in genes known to cause mitochondrial disorders (for example, *POLG*, *TPP1*, *PDSS2*, *COQ2*, etc., were all negative). In particular, no pathogenic mtDNA mutations (such as MELAS m.3243A>G) were present. Thus, secondary CoQ10 deficiency due to other mitochondrial disorders was ruled out, and primary *COQ8A* deficiency remained as the unifying diagnosis for the patient’s clinical picture. Figure 6 shows the timeline of the clinical milestones of the patient.

At presentation, the working syndrome combined cerebellar ataxia with myoclonic–tremulous movements. We first screened for acquired/metabolic etiologies (thyroid dysfunction, vitamin B12/folate deficiency, copper/ceruloplasmin/Wilson disease, hepatic/renal dysfunction, toxin or medication exposure), all of which were unremarkable. Autoimmune/inflammatory causes (autoimmune encephalitis panel, ANA/ENA, thyroid antibodies) and infectious etiologies (HIV, syphilis serology; CSF inflammatory profile if present) were not supported. Brain MRI excluded mass or vascular malformation and showed only mild cerebellar atrophy. Needle EMG did not reveal neurogenic or myopathic damage, supporting a central origin. Given the markedly reduced plasma CoQ10 and the persistent myoclonic–tremulous phenotype with cerebellar signs, primary CoQ10 biosynthetic defects were prioritized; whole-exome sequencing then identified compound heterozygous *COQ8A* variants, aligning biochemical, clinical, and genetic evidence with *COQ8A*-related disease.

## Discussion

This case expands the recognized phenotypic spectrum of *COQ8A*-related primary coenzyme Q10 (CoQ10) deficiency by highlighting myoclonic–tremulous movements as a leading manifestation in a young adult. *COQ8A* deficiency, also referred to as COQ10D4 or ADCK3-related ataxia, classically presents with childhood-onset cerebellar ataxia, seizures, and variable multisystem involvement. Our patient instead exhibited semi-continuous myoclonic tremor and truncal jerks with only mild dysarthria and ataxia, illustrating that hyperkinetic movement disorders may dominate the clinical picture even in the absence of overt epilepsy or severe ataxia. This emphasizes the importance of recognizing treatable mitochondrial disease in atypical movement disorders. The biochemical finding of low plasma CoQ10 and the identification of compound heterozygous *COQ8A* variants confirmed a primary CoQ10 biosynthetic defect. The prompt improvement after CoQ10 supplementation underscores the reversibility of neuronal dysfunction when treated early.

From a genetic perspective, more than 40 pathogenic or likely pathogenic *COQ8A* variants have been reported across ethnic groups, including missense, nonsense, and frameshift changes. In the multicenter cohort by Traschütz et al., biallelic loss-of-function mutations (e.g., two truncating alleles) were associated with pure cerebellar ataxia, whereas patients harboring at least one missense variant tended to display broader neurological involvement such as seizures, dystonia, or cognitive impairment (Traschütz et al., 2020). Similarly, Cotta et al. described early-onset ataxia and respiratory chain dysfunction in cases with novel *COQ8A* missense variants (Cotta et al., 2020). The two novel missense variants in our patient-NM_020247.5:c.641T>A (p.Leu214Gln) and NM_020247.5:c.1621T>C (p.Ser541Pro) are both located in the kinase-like domain and affect highly conserved residues, aligning with prior observations that lesions in this region can disrupt the stability of the CoQ10 biosynthetic complex and manifest as mixed cerebellar and extrapyramidal symptoms. Although precise motif-level correlations remain speculative, our findings are consistent with existing evidence that domain-specific missense changes confer greater clinical heterogeneity than truncating mutations.

Therapeutically, CoQ10 supplementation remains the mainstay of management for primary CoQ10 deficiency. Improvement in motor function, coordination, and seizure control has been reported in approximately 40%–50% of *COQ8A* cases treated with high-dose CoQ10 (10–30 mg/kg/day). High-dose oral CoQ10 supplementation is currently the most established and evidence-supported intervention for primary coenzyme Q10 deficiencies (Hargreaves et al., 2020). Although no standardized dosing regimen has been established, typical doses range from 10 to 30 mg/kg/day, which generally corresponds to 300–1,200 mg per day in divided doses for adults. Our patient was treated with 300 mg twice day of ubiquinone, which falls in the lower dose of this range, justified by his moderate presentation. After 1 year of therapy, his myoclonic tremors showed improvement. At the last follow-up, when the patient’s condition was largely stabilized on CoQ10 therapy, levetiracetam (500 mg twice daily) was introduced for symptomatic control of residual myoclonus, with good effect. The patient remained stable thereafter without further exacerbations. This favorable outcome is consistent with approximately half of the cases in the literature that report neurological improvement with CoQ10 supplementation. In the largest series of *COQ8A* patients (30 treated individuals), 43% responded to CoQ10 with objective gains in motor function or seizure control, among reported symptoms, cerebellar ataxia appears to be the most consistently responsive to therapeutic intervention (Tan and Airik, 2021).

Muscle biopsy in our case revealed no major mitochondrial pathology, consistent with previous reports that histopathological changes in *COQ8A* deficiency may be subtle or absent. The absence of cardiomyopathy or systemic involvement may relate to the relatively early therapeutic intervention and moderate disease burden. Nevertheless, our data cannot confirm a preventive effect of CoQ10 on cardiac manifestations; such inferences require longitudinal studies with larger samples. Experimental data from *COQ8A*-deficient mice have provided mechanistic insights into Purkinje cell vulnerability, yet direct extrapolation to human disease remains limited.

In this case, we report paroxysmal dyskinesias and mild coordination difficulties in the limbs with an adult onset caused by compound heterozygous variants of the *COQ8A* gene. This constellation of symptoms required careful differentiation from other neurological disorders with overlapping features. The differential diagnosis in an adolescent with paroxysmal dyskinesias and ataxia includes a broad range of conditions, such as autosomal recessive cerebellar ataxias (ARCA, e.g., *SETX*, *SYNE1* mutations), ataxia telangiectasia, organic acidemias, and juvenile-onset mitochondrial epileptic syndromes. Beyond these ataxic and epileptic disorders, it is also essential to distinguish COQ8A-related movement abnormalities from other paroxysmal movement disorders such as PRRT2-, GLUT1-, and PNKD/PED-related conditions. PRRT2-related paroxysmal kinesigenic dyskinesia (PKD) typically presents with very brief, seconds-long attacks triggered by sudden voluntary movement or startle, and often responds dramatically to low-dose carbamazepine (Alahmar, 2019). In contrast, the movements in our patient were semi-continuous, lasted minutes to hours, and were not provoked by sudden motion, arguing against a classical PKD phenotype. Paroxysmal exertion-induced dyskinesia (PED) secondary to GLUT1 deficiency typically presents withepisodes precipitated by sustained physical activity or fasting, often accompanied by low cerebrospinal fluid glucose (hypoglycorrhachia) and clinical improvement on a ketogenic diet (Haefeli et al., 2011), these features were absent in this case. Importantly, a significantly reduced CoQ10 level suggested a defect in CoQ10 biosynthesis, and the identification of biallelic pathogenic variants in *COQ8A* established the diagnosis of primary CoQ10 deficiency. Although secondary CoQ10 deficiency can occur in other mitochondrial diseases or with certain medications, the patient’s clinical picture-dominated by tremor and limb ataxia-aligned well with known presentations of *COQ8A*-related disease.

Although this report is based on a single clinical observation, several aspects strengthen its interpretive value. Functional assays were not performed, and future *in vitro* studies will be helpful to further substantiate the pathogenicity of the identified variants. The follow-up period was relatively short, and longer observation would better clarify disease trajectory and treatment durability. In addition, plasma CoQ10 measurements may not perfectly reflect tissue levels, as muscle or fibroblast analyses were not feasible in this case. Nevertheless, the consistent convergence of clinical presentation, biochemical findings, and genetic evidence provides credible support for a causal relationship between the *COQ8A* variants and the described phenotype.

In summary, this case illustrates both the devastating potential of untreated primary CoQ10 deficiency and the remarkable improvements that are possible with therapy. It expands the known *COQ8A* mutational spectrum by documenting novel pathogenic variant (p.Leu214Gln and p.Ser541Pro). Our experience aligns with the literature that emphasizes early diagnosis (preferably in childhood, before irreversible neuronal loss) and early CoQ10 supplementation as key determinants of outcome. For clinicians, primary CoQ10 deficiency should remain a key consideration in patients with unexplained ataxia or movement disorders, particularly when accompanied by cerebellar atrophy or reduced CoQ10 levels.

## Data Availability

The data that support the findings of this study are available from the corresponding authors on reasonable request.
